# Family history of bruxism: a case-control study based on the ecological momentary assessment of awake bruxism

**DOI:** 10.22514/jofph.2025.056

**Published:** 2025-09-12

**Authors:** Ovidiu Ionut Saracutu, Matteo Pollis, Luca Guarda-Nardini, Alessandro Bracci, Daniele Manfredini

**Affiliations:** ^1^Department of Medical Biotechnologies, School of Dentistry, University of Siena, 53100 Siena, Italy; ^2^Unit of Oral and Maxillofacial Surgery, Ca’ Foncello Hospital, ASL 2 Marca Trevigiana, 31100 Treviso, Italy; ^3^Department of Neurosciences, School of Dentistry, University of Padova, 35128 Padova, Italy

**Keywords:** Awake bruxism, Ecological momentary assessment, Family history, STAB, Question-naire, Genetics, Relatives, Single nucleotide polymorphism

## Abstract

**Background**: In everyday clinical practice, the screening of specific genes for awake bruxism (AB) is not a sustainable and feasible practice; most of the time, the only information that clinicians can rely on is investigating the family history of bruxism. Nevertheless, little is known about the relationship between the reported history and AB frequency. The aim of the present paper is to assess the existence of any differences in the frequency of self-reported AB behaviors between healthy young individuals with and without a positive report of family history of bruxism. **Methods**: Participants were recruited within the community of the University of Siena by advertising the possibility of taking part in the investigation through the academic website and mail. All the participants performed a seven-day monitoring of the frequency of self-reported AB via the ecological momentary assessment (EMA). Moreover, for the assessment of bruxism family history, participants were asked to fill out a short questionnaire taken from the Standardized Tool for the Assessment of Bruxism (STAB). **Results**: The final sample was composed of 117 individuals (32 males and 85 females, mean age 22.3 ± 2.3). Of the total amount of participants, 48.7% reported a positive family history of bruxism. The Mann-Whitney U test showed a statistically significant difference in the reported frequency of all AB behaviors (*i.e.*, teeth contact, mandible bracing, teeth clenching, teeth grinding) between the two groups (*p* < 0.05). Conversely, no significant difference in the frequency of AB behaviors was present between individuals with a positive report of family history in first-degree relatives compared to second-degree relatives (*p* > 0.05). **Conclusions**: Based on these findings, clinicians are recommended to not underestimate a positive family history of bruxism, as it can be an indicator of an ongoing AB.

## 1. Introduction

More than a decade ago, bruxism was reconceptualized by a panel of international experts, with separate focus on the different muscle activities and the two distinct circadian manifestations: awake bruxism (AB) and sleep bruxism (SB) [1]. Then, in 2018, an enlarged group of experts even adopted two distinct definitions [2]. Sleep bruxism was defined as a masticatory muscle activity during sleep that is characterised as rhythmic (phasic) or non-rhythmic (tonic) and is not a movement disorder or a sleep disorder in otherwise healthy individuals, while awake bruxism as a masticatory muscle activity during wakefulness that is characterised by repetitive or sustained tooth contact and/or by bracing or thrusting of the mandible and is not a movement disorder in otherwise healthy individuals. In 2023, the core members of the consensus panel published a commentary paper further specifying that bruxism, under no circumstances, can be interpreted as the disorder or comorbidity, but it can be, at most, a sign of ongoing underlying conditions [3].

In the meantime, the prevalence of AB has been far from being clarified. Systematic reviews state that it ranges between 16% and 32%, depending on the different methodologies of investigation [4, 5]. Thus, data should be taken with caution, especially in the light of the need to define the best strategies to identify awake bruxers [6]. Currently, experts in the field suggest collecting data on AB by performing an evaluation through the instrumental assessment and/or with an ecological report (*i.e.*, real time report in the natural environment) [7, 8], along with the investigation of possible etiological factors.

In this view, the Standardized Tool for the Assessment of Bruxism (STAB) [9, 10, 11] is the first multidimensional, non-stackable toolkit for clinicians and researchers that contains all the possible strategies to evaluate bruxism. It is composed of two Axes. Axis A deals with the assessment strategies of bruxism, while Axis B investigates all the etiological factors and comorbid conditions of bruxism. Regarding the instrumental assessment of AB, the STAB proposes surface electromyography (EMG) [12, 13], and ecological momentary assessment (EMA) performed with smartphone technology. While the validity of the EMG indexes has yet to be proved [14, 15], EMA is a widely used approach to gather data on the frequency of the different AB behaviors [16, 17, 18, 19, 20, 21, 22, 23].

Regarding the etiological factors (Axis B of STAB), their evaluation should be part of a comprehensive assessment of AB [24]. Many studies based on the self-report of AB and on the EMA found a consistent association with psychological factors [25, 26, 27, 28]. Moreover, a recent study showed that some specific gene polymorphisms responsible for stress-copying mechanisms are correlated with AB [29]. Indeed, the second important and non-modifiable risk factor for bruxism is represented by genetics [30], as reported by investigations that found a specific association between certain genes and AB [31, 32, 33]. However, in everyday clinical practice, the screening of specific genes for AB is not a sustainable and feasible practice; most of the time, the only information that clinicians can rely on is represented by a positive report of family history of bruxism. Nevertheless, little is known about how reliable the information on the history of bruxism is and how clinicians should interpret it. It is not clear whether people with a positive reported family history of bruxism have a higher frequency of AB compared to people without a positive history. The information on the family history is often provided directly by the patients or obtained during the anamnesis, either during a non-structured oral interview or by the administration of a questionnaire. Such information can, however, be affected by recall and knowledge bias as well as patients’ beliefs, as much as the self-report of AB [34, 35, 36].

Based on the above premises, the aim of the present study is to assess the existence of differences in the frequency of AB behaviors between healthy young individuals with and without a positive reported family history of bruxism. The study hypothesis is that there are significant differences between subjects reporting a positive and a negative family history of bruxism.

## 2. Materials and methods

### 2.1 Patients recruitment

For this study, participants were recruited within the community of the University of Siena by advertising the investigation through the university’s website and via email on 12 November 2023. To participate in the study, they had to fill out an online form with a series of criteria to verify if they were eligible. Subjects were recruited without gender or ethnic restrictions. The inclusion criteria includes being in good general health, with the absence of systemic conditions and possessing a smartphone device. The exclusion criteria includes any type of ongoing medical and dental treatment (including orthodontics) as well as a history of temporomandibular disorder (TMD) treatment and management of AB. Individuals with TMD were excluded via the online administration of the TMD Pain Screener [37]. Subjects under the effect of any pharmacological drugs were excluded as well. Such criteria were adopted to avoid the possible confounder effect of TMDs, drugs and other types of treatment on the frequency of AB. Once participants accepted to take part in the study, they signed the informed consent and received an email with the instructions to follow. Participants were asked to attend a 2-hour seminar with the leading investigator (AB) and the study supervisor (DM), where they would be instructed on the use of a smartphone-based application to report AB and a questionnaire for the evaluation of bruxism family history. The seminar was held on 15 January 2024 at the University of Siena, Department of Medical Biotechnologies.

The reporting of this study adheres to the Strengthening the Reporting of Observational Studies in Epidemiology (STROBE) guidelines [38]. All individuals gave their informed consent in accordance with the Helsinki Declaration and understood that they were free to withdraw from the study at any time. The research protocol was approved by the Institutional Review Board of the Orofacial Pain Unit, University of Siena, Siena, Italy (#0119-2023).

### 2.2 Awake bruxism assessment

In the instruction email, participants received the necessary information on where to attend the meeting with the researchers. During the seminar, they received more information on the purpose of the study and the study protocol.

During the first hour of the seminar, participants received information on bruxism and on how to make use of a smartphone-based application, BruxApp (version 2.6.4, BruxApp®, World Medical Applications Srl, Florence, Italy), for the ecological momentary assessment of awake bruxism (Item A8.1 of the STAB). One of the prerequisites for the correct use of EMA is indeed providing adequate instruction for participants [18]. The study supervisor (DM) introduced the participants to the new definition of bruxism and to the different awake bruxism behaviors (*i.e.*, relaxed jaw muscle, tooth contact, mandible bracing, teeth clenching, teeth grinding). The app was set to send a series of 20 alerts, in the form of smartphone notifications, during the day, at random times for seven days. When the individual opens the notification, she/he is required to indicate the condition that is assumed to have at the moment of the alert sound. To avoid recall bias, participants were asked to indicate the condition within five minutes. If an answer is provided after more than five minutes, it is not registered and is not part of the final report of the whole monitoring period. In case the alert sound arrived during an activity such as speaking, talking or eating, participants were instructed to ignore the alert. The followings masticatory muscle activities were shown in the app display:

(i) Relaxed jaw muscle: condition of perceived relax of jaw muscles, with mandibles kept apart;

(ii) Mandible bracing: condition of jaw muscle stiffness or tension like teeth clenching, but with teeth kept apart;

(iii) Teeth contact: condition of slight teeth contact like the teeth contact that the subject perceives when a 40 μ articulating paper (Bausch Occlusionspapier®; Bausch KG, Koln, Germany) is put between the dental arches and he/she is asked to slightly keep the teeth in contact to retain it on site;

(iv) Teeth clenching: all conditions in which teeth contacts are more marked than the above and jaw muscles are kept tense;

(v) Teeth grinding: condition in which the opposite teeth are gnashed or ground, independently by intensity and direction of antagonist teeth contacts.

The app was programmed to send alerts during specific time slots, from 08:00 to 12:00, 15:00 to 19:00 and 21:00 to 22:00, to minimize the chances of receiving alerts during meals. At the end of the seven days of monitoring, the app automatically generates a report with the mean frequency of each AB condition. In order for the report to be reliable, participants were asked to answer at least 60% of the alerts received each day (*i.e.*, 12 alerts on the total of 20 received). In case participants fail to reach such a threshold, the app extends the monitoring period to a maximum of seven further days till the threshold is reached. After the report is generated, it is automatically sent anonymously via email to the researchers, along with an identification (ID) code. The ID codes were given to participants when they were eligible for the study. Such IDs were also used by the participants to fill out the questionnaire on the family history of bruxism. The final report contains the frequencies of each AB behavior calculated as a percentage of the total alerts answered.

### 2.3 Family history of bruxism assessment

After the participants were provided with the necessary information to perform the EMA of AB, they were required to fill out a short questionnaire taken from the STAB on family history of bruxism (Item B5.1). The following question was asked:

Do you know of anyone in your family (for example, father, mother, children) who has had any history of bruxism occurrence?

- No

- Yes

- If yes: Father/Mother/Son/Daughter/Grandfather/Grandmother

- Don’t know

### 2.4 Statistical analysis

Data were extracted by two independent authors and put into an Excel document. A descriptive analysis of each awake bruxism behavior was performed. Data on AB frequency were expressed as a percentage with respect to the number of alerts answered. The frequency was calculated on an individual basis, and individual frequencies were used to calculate an average of the study population on a daily basis. At the end of the seven days of monitoring, the mean frequency of each condition was assessed both for each subject and for the entire study population. Data were reported as mean values, standard deviation (SD) and coefficient of variation (CV). Mann-Whitney U test was used to test the null hypothesis. Moreover, the same type of test was adopted to assess if there is any significant difference between subjects reporting family history among first-degree relatives (Father/Mother/Son/Daughter) or second-degree relatives (Grandfather/Grandmother). The level of significance was set at *p* < 0.05 with a 95% confidence interval (CI).

## 3. Results

A total of 160 individuals filled out the form to assess their eligibility for the study. Of them, 15 were excluded due to ongoing orthodontic treatment and 11 were positive for TMDs. 12 subjects were excluded due to the assumption of drugs. Of the remaining 122, five did not complete the monitoring period within the 14 allowed days. Thus, the final sample was composed of 117 individuals (32 males (M) and 85 females (F), mean age 22.3 ± 2.3). The composition of the sample was formed by undergraduate students (38%) (31 F, mean age 19 ± 1.1), young dentists working in the hospital setting (22%) (19 F, mean age 24 ± 2), employers of the faculty (31%) (28 F, mean age 25 ± 2.5) and post-graduate students (9%) (7 F, mean age 23 ± 0.2). Table 1 summarizes the demographic features of the participants. Of the total amount of participants, 48.7% reported a positive family history of bruxism. No subject answered “don’t know” to the question on family history. Most of the participants reported a family history of bruxism only in a first-degree relative (77%), while (23%) in a second-degree relative. No participant indicated positive family history in both first- and second-degree relatives.

**Table 1.** Demographic characteristics of the sample.

	Negative family history of bruxism	Positive family history of bruxism
Sample size	60 (51.3%)	57 (48.7%)
Age (Mean ± SD)	22.1 ± 2.1	22.9 ± 2.9
Gender	17 M (28%), 43 F (72%)	15 M (26%), 42 F (74%)

*M: male; F: Female; SD: standard deviation.*

Table 2 shows the overall mean and standard deviation (SD) of the alert response rate, while Table 3 shows the mean frequency and SD of each AB behavior as a percentage.

**Table 2.** Alert response rate for the seven days of AB monitoring in percentage.

Alert response rate	D1	D2	D3	D4	D5	D6	D7	Mean of confirmed alerts
Mean	72.3%	71.1%	77.3%	72.1%	74.4%	75.8%	71.5%	73.7%
SD	10.1%	11.1%	10.3%	17.2%	16.4%	18.4%	11.7%	7.1%

*SD: standard deviation.*

**Table 3.** Mean frequency of AB behaviors in percentage.

Activity	Mean	SD	CV	Range
Relaxed Jaw Muscles	76.4%	27.5%	0.4%	7.2–100.0%
Teeth Contact	12.6%	12.4%	1.0%	0.0–76.0%
Mandible Bracing	6.5%	13.2%	2.0%	0.0–76.2%
Teeth Clenching	3.8%	2.3%	0.6%	0.0–24.1%
Teeth Grinding	0.7%	0.2%	0.3%	0.0–2.7%

*SD: standard deviation; CV: coefficient of variation.*

In Table 4, the mean and SD frequency of AB behavior of participants that reported positive and negative bruxism family history. The Mann-Whitney U test showed a statistically significant difference in the frequency of all AB behaviors (*i.e.*, teeth contact, mandible bracing, teeth clenching, teeth grinding) between the two groups (*p* < 0.05). Based on these findings, the null hypothesis was rejected.

**Table 4.** Mean and standard deviation of the different AB behaviors between individuals with positive and negative family history of bruxism.

	Negative Family history of Bruxism (Mean ± SD)	Positive Family history of Bruxism (Mean ± SD)	NFH ≠ PFH *p*-value
Teeth Contact	8.5 ± 9.4	13.1 ± 14	0.035
Mandible Bracing	2.9 ± 3.2	7.9 ± 9.9	0.034
Teeth Clenching	0.6 ± 1.1	0.9 ± 0.9	0.0002
Teeth Grinding	0.01 ± 0.1	0.4 ± 0.2	<0.001
Total Awake Bruxism	15.7 ± 15.9	28.3 ± 21.8	0.003

*NFH: negative family history; PFH: positive family history; SD: standard deviation.*

Conversely, no significant difference in the frequency of AB behaviors was present between individuals with a positive family history in first-degree relatives compared to second-degree relatives (*p *> 0.05). The results of the statistical analysis are indicated in Table 5.

**Table 5.** Mean and standard deviation of the different AB behaviors between individuals with positive bruxism family history of first- and second-degree relatives.

	First degree relative (Mean ± SD)	Second degree relative (Mean ± SD)	FDR ≠ SDR *p*-value
Teeth Contact	10.0 ± 7.4	6.5 ± 8.6	0.35
Mandible Bracing	10.6 ± 14.1	5.6 ± 6.5	0.87
Teeth Clenching	1.1 ± 1.1	0.2 ± 2.4	0.15
Teeth Grinding	0.4 ± 0.8	0.1 ± 0.1	0.63
Total Awake Bruxism	24.6 ± 19.3	13.9 ± 13.1	0.022

*FDR: first degree relative; SDR: second degree relative; SD: standard deviation.*

## 4. Discussion

The aim of this cross-sectional investigation was to assess if there is a difference in the frequency of AB behaviors assessed via EMA between healthy individuals with and without a positive reported family history of bruxism. Results showed that individuals with a report of a family history of bruxism have a significantly higher frequency of AB compared to individuals with a negative family history. As a secondary outcome, no significant difference was present between individuals with reported bruxism family history related to first- and second-degree relatives.

The study population was composed of healthy individuals without systemic diseases. Individuals with TMDs [39] and ongoing medical and dental therapy (*i.e.*, orthodontics) were excluded since such conditions, in certain individuals, can have an impact on the frequency of AB behaviors [40, 41, 42]. Consequently, the sample size was composed of young individuals with a mean age of 22.3 ± 2.3 years.

The frequency of AB was assessed using the EMA. Such an approach gives clinicians and researchers the possibility to phenotype the different AB behaviors and study their frequency in more detail [7]. In the present investigation, teeth contact was the most frequently reported AB behavior (12.6%), followed by mandible bracing (6.5%), teeth clenching (3.8%) and teeth grinding (0.7%). The frequency of teeth grinding was negligible, and such findings are in line with the other investigations on AB performed via EMA on healthy individuals [16, 17, 18, 19, 20, 21, 22, 23].

The family history of bruxism was assessed according to the methodology proposed by the STAB, with a dichotomic question on whether subjects are aware of having a family member with a history of bruxism. In case of a positive family history, participants had to select which family member had a family history of bruxism.

To the authors’ knowledge, this is the first study that tried to compare the frequency of AB behavior between individuals with reported positive and negative family histories of bruxism via the EMA approach. The rationale for the study is based on the fact that despite it is well-known that bruxism has an important genetic component, the only way in which practitioners can assess it in everyday practice is through a simple question. Thus, the present study aimed to investigate if a positive family history of bruxism can be indicative of a higher frequency of AB assessed via an instrumental approach [34].

Due to the recent reconceptualization of bruxism into two distinct circadian manifestations, it is now evident that most of the literature on the correlation between genetics and bruxism focused on sleep bruxism only [30]. All the existing papers indicate a genetic predisposition for sleep bruxism. However, such studies have the limitation of adopting non-standardized criteria, such as the presence of attrition and tooth wear, which is not indicative of an ongoing high frequency of SB and can be caused by other factors such as gastroesophageal reflux disease (GERD) or acidic diet [43]. Indeed, a recent scoping review of the literature shows that there is still no conclusive evidence on the relationship between tooth wear severity and bruxism frequency [44]. Another important limitation of some of the past investigations regards the lack of specific DNA analysis.

Conversely, some research groups recently started to study more in detail the association between specific genes and awake bruxism. Scariot *et al*. [33] conducted a cross-sectional investigation in children between 7 and 12 years. The authors found a strong association between the single nucleotide polymorphism (SNP) *ANKK1* and awake teeth grinding. Nevertheless, it is known that the frequency of awake teeth grinding in the general population is negligible [26, 27, 28, 29, 30]; thus, the clinical relevance of such finding is doubtful. Moreover, the authors classified patients as bruxers even if they presented signs such as flattened teeth, worn tooth enamel and fractured restorations, which are not necessarily indicative of ongoing awake bruxism and could be related to other factors. Lastly, the correlation between specific polymorphism and mandible bracing was not analyzed [32]. In 2018, Oporto *et al*. [31] performed a similar investigation. The authors found that the C allele of dopamine receptor D5 (DRD5) rs6283 SNP is associated with a significant risk of awake bruxism reduction. The study was conducted on a sample of patients who had to receive treatment for bruxism. However, considering that bruxism is no longer considered a disorder that requires mandatory treatment [1, 2, 3], but a condition that is present in almost every individual up to a certain extent [26, 27, 28, 29, 30], the characteristics inclusion and exclusion criteria adopted in such study remain unclear. The authors based their assessment of AB only on self-reports by adopting a series of dichotomous questions without providing details on the frequency of the different AB behaviors. In a subsequent publication with the same methodology for the assessment of bruxism, they also found an association between the global level of DNA methylation and AB [32]. While such studies are indicative of a possible link between genetics and AB, they lack the proper assessment of bruxism, making it difficult to draw definitive conclusions on the specific relationship between AB frequency and genetics.

The strength of the present study relies indeed on the ecological momentary assessment of AB performed in a selected sample with the absence of possible confounders. Nevertheless, there are several limitations to the present observational study that prevent from generalizing the findings. The investigation was conducted on a convenience sample from the field of dentistry. On one side, it facilitated the collaboration to the data collection and the accuracy of the self-report but, on the other hand, it is not representative of the general population. Moreover, due to the lack of a multiple variable regression analysis, it was not possible to control the presence of potential confounders. Additionally, a priori sample size calculation was not feasible due to the lack of previous studies that could be used as a reference. The parent’s history of bruxism was based on the self-report, whose reliability has not been proved. Lastly, the item adopted from the STAB generically refers to bruxism without specifying if a family member has a positive history of awake bruxism, sleep bruxism or both. Based on this consideration, it could be advisable to implement the STAB with a series of items based on the type of bruxism occurring in the family and on the way in which bruxism was assessed. Indeed, the STAB is an ongoing open project that can be reviewed from time to time.

Further investigations should consider performing the EMA in both healthy individuals and parents to assess if they have similar AB frequency. Employing EMA would enhance the construct validity of measuring awake bruxism frequency in family members. In this regard, a recent pilot by Stanisic *et al*. [45] study found a similar frequency of AB in a sample of 10 young adults and 10 young parents, with no significant differences between the two groups. The sample size is, however, too small to extend such considerations to the general population. On the other hand, investigations that are performed on the assessment of specific polymorphism should take into consideration the possibility of adopting an instrumental assessment of bruxism, focusing the analysis on the frequency of certain masticatory muscle activities rather than on their presence/absence [6]. However, to better assess the hereditary factors in awake bruxism frequency, paired-sample analyses should be employed.

## 5. Conclusions

The present study found a significantly higher frequency of ecologically reported awake bruxism behaviors in individuals with a reported family history of bruxism. The difference was not significant between individuals with first- and second-degree relatives with a reported family history of bruxism. Based on these findings, clinicians are recommended to not underestimate a positive family history of bruxism, as it can be an indicator of an ongoing AB.
